# 10.1 STEM CELL-DERIVED IN VITRO MODELS FOR DEPICTING THE ROLE OF GLIA IN SCHIZOPHRENIA FROM A PROTEOMIC PERSPECTIVE

**DOI:** 10.1093/schbul/sby014.034

**Published:** 2018-04-01

**Authors:** Juliana Nascimento, Daniel Martins-De-Souza, Veronica M Saia-Cereda, Flavia C A Gomes, Stevens K Rehen

**Affiliations:** 1 University of Campinas; 2 Federal University of Rio de Janeiro; 3 Instituto D’Or de Pesquisa e Ensino (IDOR) and Federal University of Rio de Janeiro

## Abstract

**Background:**

A number of basic and translational studies have clearly indicated the vital role of glia in brain function and the pathophysiological mechanisms of neuropsychiatric disorders, including schizophrenia. The difficulty on studying the molecular basis of glial cells in vivo, led to the development of animal models, which are considered the gold standard to this type of understanding. However, the inherent difficulties in establishing these models for psychiatric disorders and the simplicity of in vitro models, especially given the recent advances in stem cell-based technologies have driven the development of sophisticated in vitro models, which may be attractive for studying the molecular basis of schizophrenia.

**Methods:**

Here, we report our investigations in terms of proteome while establishing protocols to generate human pluripotent stem cells-derived cerebral organoids as well as human cerebral organoids-derived astrocytes and oligodendrocytes.

**Results:**

The proteome of cerebral organoids show major proteins from neuronal cells as expected, but also several glial markers, supporting the notion that glial cells may be obtained out of these organoids. Besides, the proteome of three schizophrenia and three control organoids have been investigated. Proteins found are broadly distributed on functional activities such as cell growth and maintenance, energy metabolism and cell communication and signaling, and are correlated to cortical brain tissue. We also succeeded in isolating astrocytes out of cerebral organoids. These cells are under investigation in terms of molecular differences associated to schizophrenia.

**Discussion:**

The generation of brain organoids and isolation of astrocytes and eventually oligodendrocytes hold great potential for the investigation of the role of glia in schizophrenia, providing an useful approach to drug screening and disease modeling, as our results showed in schizophrenia- and control-derived cells. Additionally, proteomics adds knowledge about information and connections being formed into these models.

